# Traces of history conserved over 600 years in the geographic distribution of genetic variants of an RNA virus: Bovine viral diarrhea virus in Switzerland

**DOI:** 10.1371/journal.pone.0207604

**Published:** 2018-12-05

**Authors:** Hanspeter Stalder, Claudia Bachofen, Matthias Schweizer, Reto Zanoni, Dominik Sauerländer, Ernst Peterhans

**Affiliations:** 1 Institute of Virology and Immunology, Department of Infectious Diseases and Pathobiology, Vetsuisse Faculty, University of Bern, Bern, Switzerland; 2 University of Applied Sciences and Arts Northwestern Switzerland, Campus Brugg-Windisch, Windisch, Switzerland, Switzerland; CEA, FRANCE

## Abstract

The first records of smallpox and rabies date back thousands of years and foot-and-mouth disease in cattle was described in the 16^th^ century. These diseases stood out by their distinct signs, dramatic way of transmission from rabid dogs to humans, and sudden appearance in cattle herds. By contrast, infectious diseases that show variable signs and affect few individuals were identified only much later. Bovine viral diarrhea (BVD), endemic in cattle worldwide, was first described in 1946, together with the eponymous RNA virus as its cause. There is general agreement that BVD was not newly emerging at that time, but its history remains unknown. A search for associations between the nucleotide sequences of over 7,000 BVD viral strains obtained during a national campaign to eradicate BVD and features common to the hosts of these strains enabled us to trace back in time the presence of BVD in the Swiss cattle population. We found that animals of the two major traditional cattle breeds, Fleckvieh and Swiss Brown, were infected with strains of only four different subgenotypes of BVDV-1. The history of these cattle breeds and the events that determined the current distribution of the two populations are well documented. Specifically, Fleckvieh originates from the Bernese and Swiss Brown from the central Alps. The spread to their current geographic distribution was determined by historic events during a major expansion of the Swiss Confederation during the 15^th^ and 16^th^ centuries. The association of the two cattle populations with different BVD viral subgenotypes may have been preserved by a lack of cattle imports, trade barriers within the country, and unique virus-host interactions. The congruent traces of history in the distribution of the two cattle breeds and distinct viral subgenotypes suggests that BVD may have been endemic in Switzerland for at least 600 years.

## Introduction

Certain infectious diseases have been known for hundreds or even thousands of years. Examples include smallpox [1], rabies [2] and foot-and-mouth disease [3]. The first two stood out because of the sudden appearance and typical disease signs, whilst rabies was known as a deadly disease of humans that had been bitten by a rabid dog [2]. Understanding and fighting such diseases started the eras of immunology and microbiology in the 18^th^ and 19^th^ centuries. In fact, in 1898, the agent causing foot-and-mouth disease virus was the first to be characterized as an animal virus [3].

This article deals with a disease that, in several regards, is the very reverse of the above. Bovine viral diarrhea (BVD) is endemic in cattle (*bos taurus* and *bos indicus*) worldwide, yet the clinical signs and the eponymous causative virus (BVD virus) were first described only 1946 in North America [4, 5]. In Switzerland, BVD was first reported 18 years later [6]. There is however no evidence that BVD was a newly emerging disease in the mid-20^th^ century. The difficulty to recognize BVD was certainly due to its highly diverse and mostly mild clinical signs that may include diarrhea and coughing, but, in rare cases, may be severe and end in death of affected animals. The pathogenesis of BVD is complex. It includes two mutually exclusive types of infection, acute-transient when acquired postnatally and persistent when initiated *in utero*. The latter differs fundamentally from other types of persistent viral infections, e.g. hepatitis C in humans [7], because it is associated with a highly specific immunotolerance to the infecting viral strain [8, 9]. Persistent BVD is initiated when a pregnant cow is infected at a time when its fetus has not reached immunocompetence. The cow is transiently infected, eliminates the virus, and becomes immune to reinfection. By contrast, the fetus fails to eliminate the infection and may develop but remains persistently infected (PI] and sheds virus for life. The endemic nature of BVD itself also contributes to the stealth appearance of BVD in the cattle population. Thus, due to a high level of herd immunity, most animals are protected due to a previous transient infection, and only few in a herd will show disease signs at the same time [10, 11].

Together with classical swine fever and Border disease viruses, the two species bovine virus diarrhea virus-1 (BVDV-1) and BVDV-2 belong to the genus *Pestivirus* of the family *Flaviviridae* [12, 13]. Recently, a revised taxonomy for the genus *Pestivirus* was proposed [14] BVD viruses may infect also other species of the order *Artiodactyla*, including sheep, goats, wild ruminants, pigs and camelids [15–17]. Numerous reports have shown that especially BVDV-1 are genetically highly diverse, with different spectra of at least 21 subgenotypes of BVDV-1 reported in different countries (for a review, see [18]). Determining the genetic spectrum of bovine pestiviruses in a cattle population is difficult because transient infection is of short duration and typically only 1% of all animals are PI [10, 11]. The latter represent a “living virus repository” of the full spectrum of BVDV in a host population, but, for obvious reasons, representative sampling of these animals in a research program of its own would be challenging. Testing of all cattle for virus was made mandatory in the Swiss BVD eradication program. This enabled us to obtain and analyze samples from 36% of all PI animals detected during the first four years of this program. The original aim of our project was to establish a data bank that would link viral sequences with data of the host animals from which the viral samples had been obtained. The combination of tracing cases by analyzing viral sequences with following individual animals of interest by classical epidemiology was designed to increase the efficiency of the national BVD eradication campaign. The retrospective analysis of 7,468 cases revealed unexpected insights into the history of BVD in Switzerland. We present evidence that BVD may have been endemic in the Swiss cattle population for at least 600 years.

## Materials and methods

### Specimens and associated data

Persistent infection with BVD virus is defined as virus demonstrations in two specimens taken from an animal at an interval of >20 days. All information pertinent to the analysis of each sample and its PI host animal were compiled in a data base. Among others, each data set included the nucleotide sequence of the virus, birth date, sex and breed of the PI animal, its paternal and maternal descent and location in different farms from birth to the time of sampling. For details of the data base, see [19].

### RNA isolation, RT-PCR and nucleotide sequencing

All procedures were done exactly as described in detail in the Materials and Methods section in [19]. Briefly, RNA extraction was performed from samples of anticoagulated blood using the Qiagen BioRobot Universal Platform (Qiagen Instruments AG, Hombrechtikon, Switzerland) with the QIAamp Virus BioRobotMDx Kit (Qiagen) according to the manufacturer’s instructions. The subsequent RT-PCR was done with the QIAGEN OneStep RT-PCR Kit (Qiagen) according to the manufacturer’s instructions using the primers described in [20]. The PCR products combined with the same primer mix as used for PCR amplification were sent to Microsynth AG (Balgach, Switzerland) for Sanger sequencing.

### Analysis of data

The raw sequencing data were assembled using the SeqManII sequence analysis softwares (DNASTAR Inc. Madison, USA). The species of pestivirus and the subgenotypes were determined using the Clone Manager 9 Professional Edition (Scientific & Educational software, Cary, USA) and the MEGA version 6 [21]. Sequences obtained from GenBank were used for the assignment of the strains to a pestivirus species as described in[19] (For the complete list, see S1 Appendix).

### Statistical analysis

We used the NCSS 2007 (Kaysville, UT, USA) and GraphPad (www.graphpad.com) softwares for statistical analysis of the data. Odds Ratios were calculated using the Odds Ratio and Proportions Calculator from the Calculators sub-menu of the Tools menu in the NCSS 2007. To compare the medians of three or more groups we used the non-parametric one-way ANOVA test (Kruskal-Wallis) for data without normal distribution. To compare proportions of three or more groups we used Cross Tabulation and the Chi Square test. The Mantel-Haenzel test was used to compare the proportions of genotypes of two groups.

## Results and discussion

### The genetic spectrum of BVD viruses in the Swiss cattle population is narrow

Due to the economic impact of BVD [22, 23] control programs have gained momentum in various parts of the world [24, 25], with the Scandinavian countries being the pioneers in these efforts [26]. These programs have shown that detection and elimination of all PI animals, combined with zoosanitary measures, results in the eradication of BVD [24]. Since most animals acquire the infection from a PI herd mate, previous successful BVD eradication programs had targeted the search for PI animals to herds with a high percentage of animals seropositive to BVD virus [27, 28]. In Switzerland, studies had shown a high average herd immunity, with 60–80% of the cattle population seropositive to BVD virus and around 1% PI [29, 30]. Therefore, it was decided to test the entire cattle population for virus in the first year of the program, followed by all newborn calves in the three subsequent years. This testing schedule enabled us to obtain virus samples from 7,468 PI animals, which corresponds to 36.4% of all cattle diagnosed as being infected of 3.85 million tested during the first four years of the program (Table 1). The ratio of 85% females to 15% males seen in the first year of the program reflects the management practice of keeping females for breeding and milk production but using most males for veal and beef production at a relatively young age. The decrease in the mean and median age and the change to an even ratio between males and females was accompanied by a decrease in PI animals detected, indicating the progress of the eradication program. The success of the program was also evident in the decrease of the prevalence from 1.3% at the beginning of eradication to 0.02% four years later [31].

**Table 1:** Animals and virus strains.

year of eradication program	animals in population (n)	animals sampled (n)	virus strains analyzed (n)	PI: age [d] (range)	PI: age [d] (mean)	PI: age [d] (median)	male / female (%)
2008 (animals bornbefore Oct. 2008)	1,604,287	1,493,404	3,771	1–3525	451	339	15 / 85
2009 (animals born before Oct. 2009)	1,597,484	711,009	2,061	5–777	39	27	46 / 54
2010 (animals born before Oct. 2011)	1,591,233	719,648	1,027	3–365	24	19	50 / 50
2011 (animals born before Jan. 2012)	1,577,407	937,717	609	1–139	20	10	48 / 52
2008–2011 (Total)	n.a.	3,855,814	7,468	n.a.	n.a.	n.a.	n.a.

d: days

n.a.: not applicable

**Fig 1:**
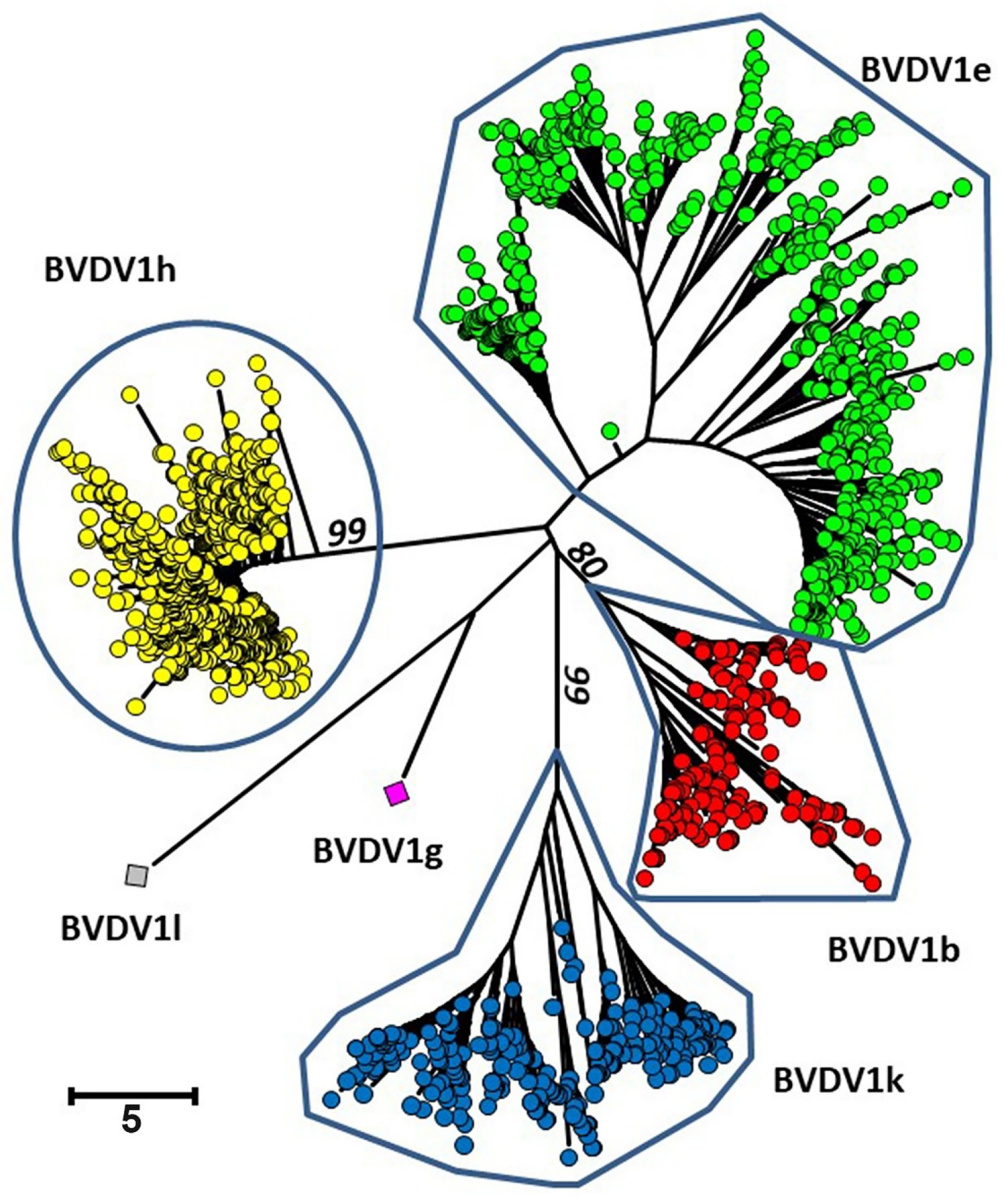
Phylogenetic analysis based on the 5’UTR of the BVD viral genome. The alignment was performed with the program MUSCLE [38] and the phylogenetic analysis with the program MEGA6 [21]. Duplicate sequences were eliminated. The evolutionary history was inferred using the Neigbor-Joining method [39]. The percentage of replicate trees in which the associated taxa clustered together in the bootstrap test are shown next to the branches (only values higher than 80 are shown) [40]. All positions containing gaps and missing data were eliminated. The classification was adapted from [18].

The different pestivirus species and subgenotypes are routinely identified by the sequence of the 5’ UTR region [32]. Of 7,468 virus strains sequenced, 49.05% belonged to the subgenotype BVDV-1h, 30.28% to -1e, 11.58 to -1k and 8.96% to -1b (Table 2 and Fig 1). Of the remaining 15 strains, one each belonged to BVDV-1g and -1l and 13 were Border disease viruses, the sheep virus known to infect also cattle [33, 34](for the GenBank accession numbers, see S1 Table). The same major four BVDV-1 subgenotypes had been found in earlier investigations with limited animal numbers [35, 36]. The two cows from which the single BVDV-1g and 1l strains originate lived in two separate remote areas of the Alps. Although antibodies to pestiviruses were found only in 1.7% of 1,877 serum samples of wild ruminants [37], chamois, ibex and red deer cannot be excluded as the origin of these two viral strains because sampling is by hunting only and many of the colonies of these animal species are sequestered from others by high mountain ranges.

**Table 2:** Subgroups of BVD viruses.

Year of eradication program	BVD virus Subgenotype (%)						BD virus (%)	Sequences (% of total)
	B	e	h	k	g	l		
**2008**	9.49	31.85	47.47	11.11	0.03	0.03	0.03	50.50
**2009**	10.43	30.62	46.92	11.98	0	0	0.05	27.60
**2010**	6.04	25.24	55.85	12.57	0	0		13.75
**2011**	6.95	25.90	55.59	10.55	0	0		8.15
2008–2011*	8.96	30.28	49.05	11.58	0.01	0.01	0.11	100.00

* weighted average, i.e., average of total of strains

### The BVDV-1 subgenotypes are concentrated in different regions

**Fig 2:**
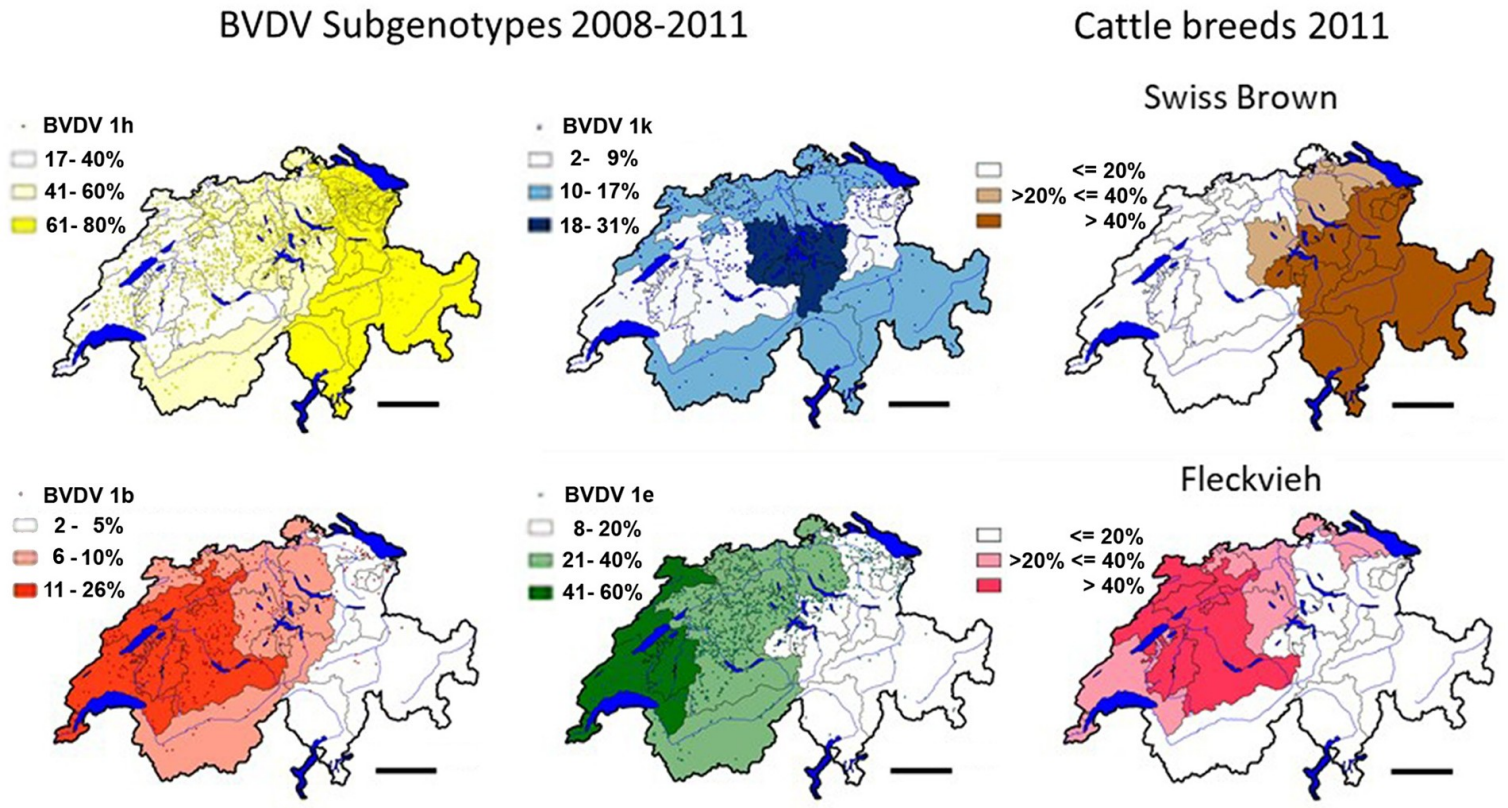
Distribution of the main subgenotypes of BVDV-1 and of Swiss Brown and Fleckvieh cattle. The minimal and maximal proportions (in %) per canton between each of the four main subgenotypes BVDV-1b, -1k, -1e, and -1h was divided in three equal fractions and color-coded with the lowest third drawn in white, the middle third being light-colored and the highest third drawn in dark color. The percentage of the fractions per subgenotype are indicated in Fig 2. The dots represent the location of individual PI animals. The distribution per canton of the Fleckvieh and Swiss Brown cattle breeds are shown, with less than 20%, between 20% and 40%, and more than 40% indicated in white, light-brown and light-red, and dark brown and dark red, respectively. Data of cattle were taken from the national animal movement data base, populations as of 31^st^ December, 2011 [41]. For details of the borders of individual cantons, see S1 Fig. The maps were calculated and drawn with the quantum GIS version 1.80-Lisboa software [47]. Bar = 50km.

We compiled a data bank that links the 5’ UTR sequence of every virus strain with a wide array of detailed information of its host animal [19]. The latter was available from the national animal movement data base in which data of every animal are recorded, among others, the age, sex, maternal ancestry and offspring, and locations in different farms throughout life until death [41]. The combination of sequence and host data proved very useful because this enabled us to trace unclear chains of infection starting from the side of the virus as well as tracking individual animals of interest [19]. On average, the information on 7,468 PI animals and virus strains accumulated over four years corresponds to one data point per 2.04 square kilometers of the agricultural area of Switzerland (15,227 square kilometers) [42]. This made it possible to plot high resolution maps of the locations of PI animals and their viral strains. One dot in Fig 2 represents one or more PI animals in individual farms. The intensities of the underlying colors mark cantons with high, intermediate and low concentrations of viral strains of a given subgenotype. Note that the densities are relative, rather than absolute, because the numbers of strains of the four subgenotypes are different. For example, the low density of the strains of subgenotype BVDV-1h corresponds to a higher density in the less frequent subgenotype BVDV-1b (compare also with Table 2). The highest densities of the subgenotypes BVDV-1b and -1e were detected in Western Switzerland, with an extension to the North-East, whereas the highest concentrations of BVDV-1h were observed in the Eastern part of the country. Strains of the BVDV-1k subgenotype were concentrated in the central Alpine region and extended to the Midland between the Alps and the Jura mountain ridge and to the Valais in the South-West, the Ticino in the South, and to Graubünden in the South-East (see S1 Fig for a map of the cantons). Strains of BVDV-1b, -1e and -1h subgenotypes were reported also elsewhere in Europe (reviewed in [18]). By contrast, the BVDV-1k subgenotype was observed only in very low numbers outside of Switzerland, in Austria [43, 44], Italy [45], Southern Germany [32] and Spain [46].

### The four BVDV-1 subgenotypes are associated with two traditional cattle breeds

The clear difference in the geographic location of the BVDV-1b, -1e, -1h and -1k viral strains guided us to a research field well beyond that of tracing individual chains of infection. Specifically, we searched if being PI with strains of different subgenotypes was correlated with any of the host properties recorded in the animal data bank [19]. This search revealed a clear correlation between the subgenotypes BVDV-1b and -1e with cattle of the Fleckvieh breed, and of BVDV-1h and -1k with the Swiss Brown cattle, the two main traditional Swiss cattle breeds. The odds ratio of being PI with the subgenotypes BVDV-1h or -1k was 3.44 in Swiss Brown vs. 0.291 in Fleckvieh. It is important to point out here that this does not imply a difference in susceptibility, but reflects historic developments, conservative husbandry and unique aspects of the BVD virus-host interaction that will be addressed in detail below. Moreover, arguing against sampling errors, the ratios of sequences analyzed from PI animals to the total populations of the two cattle breeds were similar (S2 Fig), as were those of PI animals analyzed from the 26 Swiss cantons (S3 Fig; for a map of the cantons, see S1 Fig). This resulted in largely congruent distribution maps of the subgenotypes BVDV-1b and -1e with the Fleckvieh populations, and those of BVDV-1h and -1k with the Swiss Brown cattle breed (Fig 2).

### The distribution of the two cattle breeds can be traced back to distinct historic events

**Fig 3:**
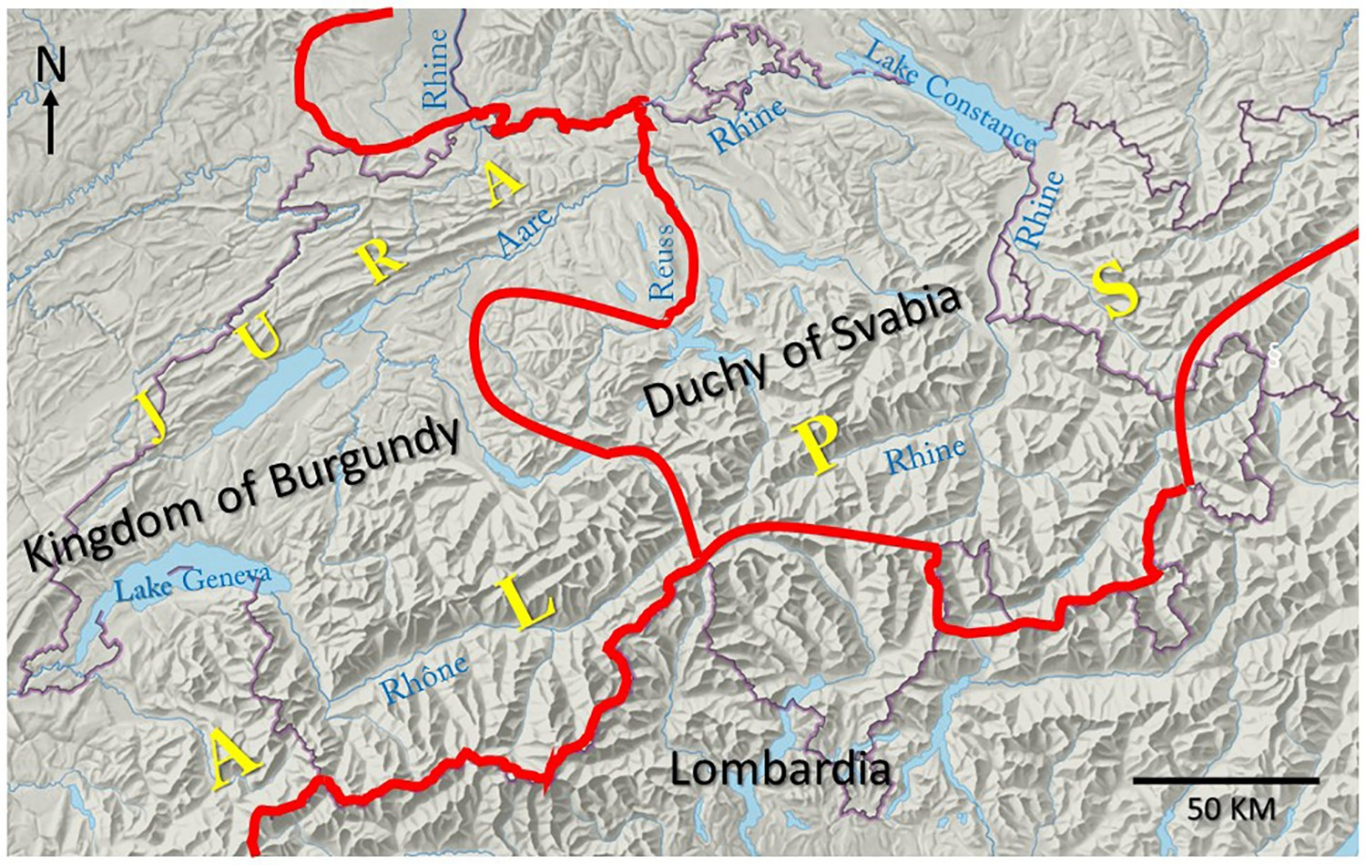
Political constellation in Central Europe, early 10^th^ century. The approx. borders between the Kingdom of Burgundy, Duchy of Svabia and Lombardia are indicated in red color. The Rhine, Rhône, Reuss and Aare rivers have their origin in the central Alps of Switzerland. The Rhine river runs through Lake Constance and continues in a northerly direction to the North Sea. The Rhône river runs through the Valais (S1 Fig), continues through Lake Geneva and then flows through the Rhône valley before joining the Mediterranean Sea. The Reuss river flows into the Aare river, which then joins the Rhine river.

**Fig 4:**
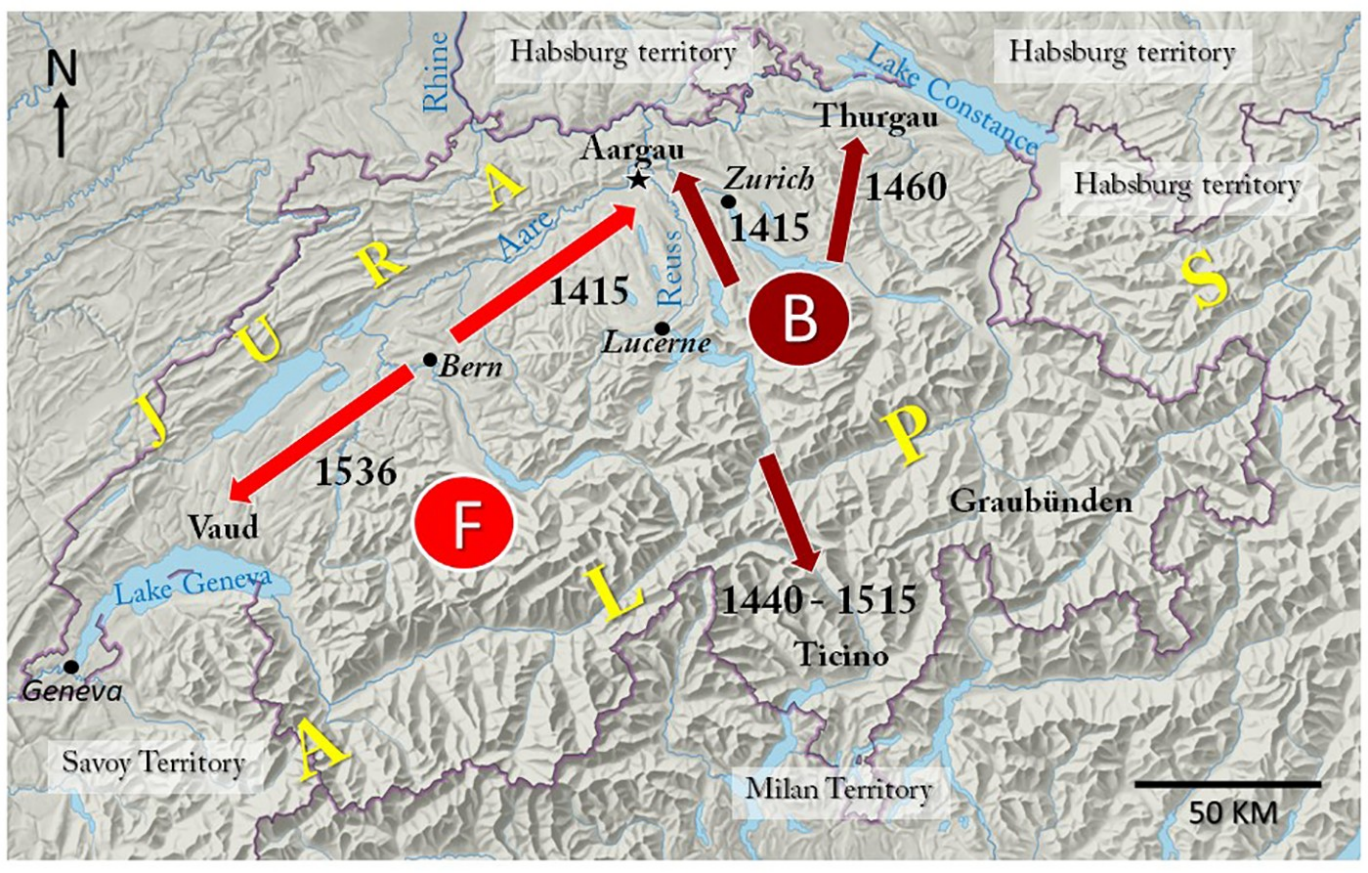
Major expansion of the Swiss Confederation in the 15^th^ and 16^th^ centuries. Shown are the major directions of expansion of the Bernese (red arrows) and of the other Confederate States (brown arrows). The location of the ancestral castle of the Habsburg dynasty is indicated by a black asterisk. **B** shows the origin of the Swiss Brown cattle breed in the central Alpine region and **F** that of the Fleckvieh breed in the Bernese Alps. For the current borders between the cantons, see S1 Fig.

Animals of both cattle breeds are found in the Alpine region, in the adjacent Midland North of the Alps, and in the Jura ridge that runs E to W along the borders to Germany and France (S1 Fig). Hence, climatic differences as the cause of the distribution of Fleckvieh and Swiss Brown cattle can be ruled out. The search for an explanation of this peculiar geographic distribution directed us back in time to a political constellation that existed in the 10^th^ century. What might be called the “Fleckvieh region of Switzerland” was then part of the Kingdom of Burgundy, whilst Swiss Brown is found mostly in the region that belonged to the Duchy of Svabia (compare Fig 2, cattle breeds, with Fig 3) [48, 49]. However, although largely congruent with the political zones of influence of the Kingdom and the Duchy, it is unlikely that this ancient political constellation itself directly determined the current distribution of the two traditional Swiss cattle breeds. Firstly, domesticated cattle of the *bos taurus* species arrived in Switzerland as early as 5,000 years BC [50], and, secondly, in early medieval time, cattle breeding was concentrated in the Alps, whereas arable farming dominated in the other regions [51]. The earliest records dating back to the 13^th^ and 14^th^ centuries indicate that Fleckvieh has its origin in the Western Bernese Alps (marked “F” in Fig 4) [52], whereas the central Alps are the home of Swiss Brown (marked “B”) [53]. The expansion from these Alpine origins to the other parts of the country is closely linked to conflicts of the early Swiss Confederation with the Dukes of Habsburg, Savoy and Milan. A dramatic major expansion of the Confederate states was triggered at the Council of Constance (1414–1418), a time when both the Swiss Confederation and the Duchy of Habsburg were part of the Empire of German Nation that was ruled by an elected King [54]. To punish the Duke of Habsburg for taking sides with one of three popes who competed for supremacy, King Sigismund invited the Swiss to seize the Duke’s land in Aargau, where also the ancestral castle of the Habsburg dynasty is located (indicated by black asterisk in Fig 4). In the spring of 1415, Bernese troops swiftly conquered Habsburg territory in an easterly direction along the river Aare and in the West of the river Reuss, while the Confederates from the central Alpine region, Zurich and Lucerne seized land East of the river Reuss [55]. In 1460, Habsburg territory located in the West of Lake Constance was conquered by a coalition of Confederates from the central and Eastern regions [56]. The Italian-speaking Ticino in the South of the Alps was taken by the Confederates of central Switzerland from the Dukes of Milan in several steps from 1440 to 1515 [56], and in 1536 the Bernese seized land located east of lake Geneva from the Duke of Savoy [57].

After these political upheavals, the imperial City state of Bern and its dependent territories extended over a large part of the former area of the Kingdom of Burgundy in Switzerland, while the other Confederates held mostly land that had been part of the Duchy of Svabia. This territorial constellation and the political system of the Swiss Confederation were instrumental for the geographic distribution of the two cattle breeds, and also for its conservation. The Confederate states were sovereign and conceded only limited powers to the Swiss Diet (referred to as “Tagsatzung”). A high level of independence was the case not only in politics, but also in economic matters. Accordingly, cattle from the Bernese Alps spread in the Bernese territories and in the adjacent Northern Border regions to France and Germany, whereas cattle from the central Alpine region spread in the territories held by the other Confederates and to Italy (Fig 4). Trade between the areas of the Confederate states was limited by multiple barriers, including custom duties, different currencies and jurisdictions [51]. It was restricted also by a complex social system that included serfdom and subjects and often cattle were not owned by the farmers but belonged to wealthy persons living in the imperial cities [51, 58].

While the densities of the BVDV-1b and -1e subgenotypes largely overlap in the area where animals of the Fleckvieh breed dominate, it is obvious that the BVDV-1h and -1k subgenotypes are associated with two distinct Swiss Brown regions (Fig 2). This supports an earlier suggestion that the Swiss Brown cattle population of Eastern Switzerland may have roots also in Italy [53]. Moreover, it is consistent with the fact that Graubünden, the largest canton that covers most of the South-Eastern area where BVDV-1h dominates, had close trade ties with Northern Italy and joined the Swiss Confederation only in 1803.

**Fig 5:**
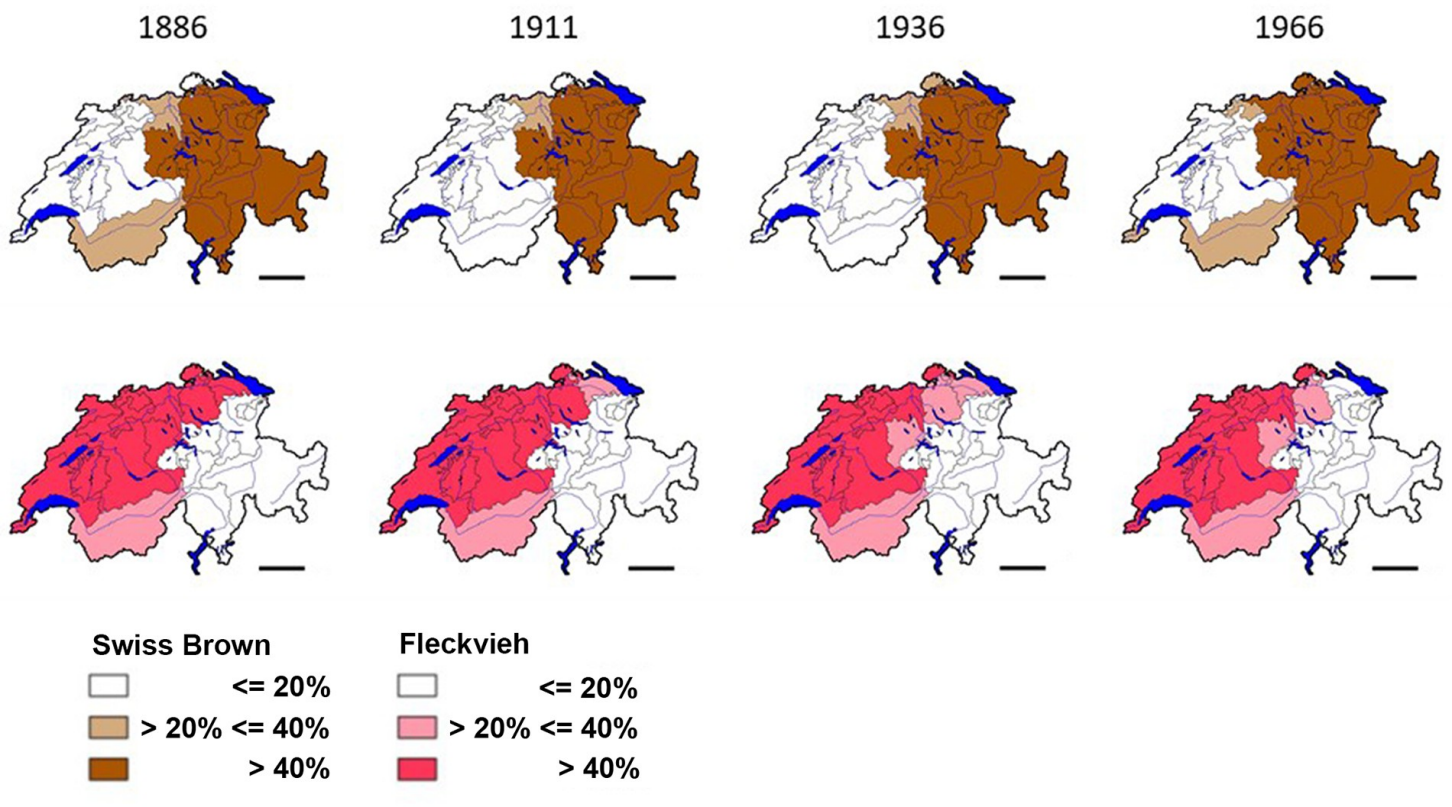
Distribution of Swiss Brown and Fleckvieh cattle from 1886 to 2011. The distribution per canton of the Fleckvieh and Swiss Brown cattle breeds are shown according to the same criteria as in Fig 2. Data for 1886, 1911, 1936 and 1966 are taken from annual yearbooks [59]. Data of the year 2011 are as described in Fig 2 [41]. Bar = 50km. The maps were calculated and drawn with the quantum GIS version 1.80-Lisboa software [47].

The old Swiss Confederation and its political, social and economic system ended in 1798 when Switzerland became a theatre of war between France and the allied European monarchies. By rulings of Emperor Napoleon I of France, the dominant Confederate states lost their dependent territories. During the 19^th^ century the rather loose alliance of Confederate states became more centralized, with the introduction of a common currency, the abolishment of trade barriers between the cantons and the Swiss federal authorities gaining more power. These profound political changes however occurred in a stepwise fashion over a time of 80 years. The first official Swiss cattle census of 1886 showed that the geographic distribution of Fleckvieh and Swiss Brown still reflected the political constellation of the pre-Napoleonic era. Though the size of the cattle population fluctuated between 1.2 and 1.8 million in the last 125 years, the distribution of, and ratio between, the populations of the Fleckvieh and Swiss Brown cattle breeds had not changed by 2011 (Fig 5).

### The interplay between husbandry and biology preserves the genetic spectrum of BVD viruses in the cattle population

Historical developments determined the current distribution of the Fleckvieh and Swiss Brown cattle and explain its conservation over time. Unknowingly, humans also influenced the diversity of BVDV viruses in the Swiss cattle population. As shown above, the genetic spectrum of bovine pestiviruses was very narrow, with two subgenotypes associated with each of the two cattle breeds. Although the size of the Italian cattle population is only 5 to 5.5 million, the genetic diversity of pestiviruses in Italy is very high, with BVDV-2, HoBi-like pestivirus, and some 18 different subgenotypes of BVDV-1 [45, 60, 61]. This high level of diversity can be explained by a long tradition of importing large numbers of cattle of unknown BVD status from different countries. The estimate for PI animals imported to Italy between 2003 and 2010 stands at 1,200 to 16,000 head per year, depending on a calculated prevalence of 0.1% and 1.5%, respectively (S1 Personal Communication. Luzzago and Cerutti). By contrast, there is no evidence that cattle were imported in Switzerland in any substantial numbers. In 1966, Fleckvieh and Swiss Brown cattle accounted for 96.7% of the total cattle population [59]. Before the mid-1960s importation of cattle to Switzerland was essentially prohibited and new breeds started from few imported females and grew slowly, with imported semen. In our study, viral strains found in PI Fleckvieh and Swiss Brown cattle accounted for 56% of all analyzed, followed by some 15% of strains from PI animals of mixed breeds (Kreuzung) and 10% of Holstein (S2 Fig).

The profound introgression since the late 1960s of Fleckvieh by Red Holstein and of the original Swiss Brown by Swiss Brown from the USA and Canada was by semen and apparently did not result in the introduction of “exotic” BVD virus subgenotypes, such as BVDV-1a that is numerous in North America [18]. The population of Holstein cattle is the most numerous of the newly introduced breeds (S2 Fig). The spectrum of subgenotypes of the viral strains obtained from PI Holstein cattle was close to those of the other PI animals in the same cantons (S4 Fig). This indicates that Holstein PI animals had been infected by cattle of other breeds in the same regions.

Although in our population the ratio of PI to uninfected cattle was equal at birth between males and females, males only accounted for some 15% of the total population in the first year of the eradication program (Table 1). PI bulls were hardly ever used for breeding even before the advent of artificial insemination because BVD virus negatively impacts male fertility [62, 63]. However, females were not only more important than males in the epidemiology of BVD because of their higher numbers, but also because only females can generate PI offspring. Mostly female cattle are traded, which may result in BVD virus introduction in a herd by a PI animal, or by a “Trojan cow” pregnant with an infected fetus. By far most trading was, and still is, within the same breed, which resulted in conservation of the genetic spectrum of dominant BVDV-1 subgenotypes. The strong tendency to conserve the local spectra of the subgenotypes of BVDV-1 was evident also from the movement data of the dams that had given birth to PI calves. Of these dams (information available from n = 7,119), 31% were still at the same farm, 55% had moved to a farm in the same canton and only 14% had moved to another canton at the time when their PI offspring was detected. Transient infections with BVDV-1 confer lifelong immunity and fetal protection [64, 65] against strains of different BVDV-1 subgenotypes [66]. Before the beginning of the eradication program, 60–80% of all animals were immune, some 40% already when 12–24 months old [29, 30]. No herds were completely seronegative [30]. Hence, the high level of immunity in the cattle population decreased the chances of any newly introduced “exotic” subgenotypes of getting a foothold and spreading in the population.

As an additional important aspect contributing to conservation of the spectrum of breed-associated subgenotypes, the four subgenotypes displayed a similar fitness. The age-distribution of cattle PI with strains of the four subgenotypes was not different, and, regardless of the subgenotype, we detected PI animals that were over eight years old and had given birth to several calves that were also PI (S5 Fig). Moreover, in acutely infected animals, severe disease was very rare [67]. Experimentally, it was possible to reproduce a severe disease with such strains [68]. However, they belonged to the same subgenotypes already present [67] and never established new chains of infection of a more virulent phenotype under field conditions. This indicates that the fitness of BVDV can largely be defined as the ability to be transmitted efficiently to susceptible animals without causing severe disease during transient infection, and to establish persistent infection in fetuses without causing elevated rates of abortion or lowering the average lifespan of PI animals. Overall, these observations indicate that the four subgenotypes existed in a steady-state equilibrium in the cattle population. Hence, the uneven geographic distribution of the four BVDV-1 subgenotypes was unrelated to a difference in the virus-host interaction.

### Molecular clock vs. history?

We showed in the preceding sections how distinct historic conflicts that date back 600 years determined the geographic distribution of Fleckvieh and and Swiss Brown cattle and how trade restrictions and traditional husbandry practices enhanced the key role of females in preserving the genetic spectrum of BVDV in the two cattle populations. The wide knowledge of the role of humans in the history of the two cattle breeds in Switzerland is in stark contrast to the complete lack of information of the history of BVD and its eponymous virus prior to the first description in North America [4, 5].

In the absence of archeologic samples, the age of viruses may be calculated, using the concept of the molecular clock [69]. For bovine, porcine and ovine pestiviruses, the time of divergence from a common ancestor was calculated to the year 1483, with a wide margin for the highest posterior density HPD_95_ of 600 to 1892. The emergence of BVDV-1 and BVDV-2 from a putative ancestor was calculated to a time between 1629 to 1736 [70], and that of the emergence of BVDV-1b and -1e to a time between 1889 to 1950 and 1957 and 1988, respectively [61].

However, calculations of the age of RNA viruses have been criticized, both on theoretical considerations and on epidemiological evidence. In the absence of virus samples that date back hundreds or even thousands of years, calculations of emergence times must depend on virus samples obtained during a very short period [71], which decreases the precision of extrapolations from sequence comparisons. Furthermore, such calculations do not take into account a stabilizing effect of virus-host co-speciation [72] and changes related to virus transmission between different host species [73]. It was also argued that molecular clock calculations ignore the effects of the quasispecies nature of RNA viruses [74] and of geographic information of virus samples [75]. The molecular clock concept also fails to explain epidemiological observations, such as the global presence of genetically diverse variants of hepatitis C virus, a *Hepacivirus* of the *Flaviviridae* infecting humans and of Hepatitis B virus [72, 76]. In the case of the latter, the discrepancy between calculated and real emergence time was directly demonstrated by the analysis of virus specimens from a 16^th^ century mummy [77]. Emergence and spread of the highly diverse bovine pestiviruses [18] in the short time frame suggested by molecular clock calculations [61, 70] is unlikely, because different species and subgenotypes of pestiviruses are present in taurine and indicine cattle and in other even-toed ungulates all over the world, often in geographically isolated regions [15]. Evidence for a strict separation of the ancestral populations of Fleckvieh and Swiss Brown cattle was recently provided by a report on genetically different strains of *Bacillus anthracis* in the regions of the two cattle breeds [78]. The congruence of the maps of the breed-associated BVDV-1 subgenotypes with those of Fleckvieh and Swiss Brown cattle observed by us strongly argues against a spread of BVDV after the expansion of the two host populations from their origins in the Alps. Such a scenario would require invasion by the four BVDV-1 subgenotypes (or their ancestors) from the surrounding countries at about the same time, followed by a spread at a similar speed in the current regions of the two cattle breeds. This would seem unlikely, especially with the BVDV-1k subgenotype, which is numerous in the central Alps, but rare outside Switzerland [32, 43–46]. As discussed with related RNA viruses [72, 76] BVDV and other pestiviruses however may be much older than suggested by calculations based on the concept of the molecular clock. The same may be said for viruses causing dramatic diseases, such as rabies [2] and foot-and-mouth disease [3], with oldest records dating, respectively, from the 18^th^ century BC and the 16^th^ of our era. In this work, we used a novel approach to trace back in time BVD, a viral disease with stealth characteristics that explain its late discovery in the mid-20^th^ century. We combined input from different areas, including viral sequence analysis, virus-host interactions and epidemiology, with areas seemingly unrelated to virology, including economy, husbandry and history. A similar approach may prove useful also to learn about the distant past of other viral infections.

## Conclusions

Here, we showed that two different pairs of subgenotypes of BVDV-1 were associated with Fleckvieh and Swiss Brown cattle. The clearly different geographic distribution of these two traditional cattle breeds can be traced back to distinct historic events of the 15^th^ and 16^th^ centuries. Overall, the close links between the history of cattle breeding with the history of Switzerland, the important role of females in BVD biology, epidemiology and husbandry support the view that genetically different subgenotypes of BVDV-1, or their ancestors, may have been endemic already in the two different cattle populations in Alps from where these breeds originated. The traces of history may have been preserved in the breed-associated BVDV-1 subgenotypes in the Swiss cattle population for at least 600 years, by a combination of human factors that acted on the cattle (political stability, very rare trade of heifers and cows between the two regions, no cattle imports from other countries) and biological factors of the interaction between BVD virus and its hosts, including a similar fitness of the four virus subgenotypes and a high cross-protective herd immunity that favorized the dominant subgenotypes of BVDV-1 in each cattle breed.

## Supporting information

S1 Appendix Sequences used for assignment of the strains to pestivirus species and subgenotypes. (DOCX)

S1 Table GenBank^(a)^ accession numbers of strains analyzed. (DOCX)

S1 Personal Communication Luzzago and Cerutti. (DOCX)

S1 Fig Cantons of the Swiss Confederation. The Swiss Confederation consists of 26 cantons. Note that some of the historic borders between the cantons differ from the current borders indicated in this Figure. Abbreviations: BS: Basel City; BL: Basel Country; SO: Solothurn; OW: Obwalden; NW: Nidwalden; SH: Schaffhausen; AI: Appenzell-Innerrhoden; AR: Appenzell-Ausserrhoden. For a complete list of the cantons, see S3 Fig.
(TIF)

S2 Fig Proportion of the different cattle breeds in Switzerland and of the sequences of BVD viruses from PI animals per cattle breed. Cattle of different breeds are indicated as a percentage of total cattle population (black bars), and the percentages of BVD viral sequences (grey bars) analyzed per PI animal detected in a given breed between 2008 and 2011 in relation to total of Switzerland are shown. “Kreuzungen” are mostly beef cattle crossbred from different breeds. In addition to Swiss Brown and Fleckvieh, Simmental, Eringer, Hinterwälder, Grauvieh and Evolène are considered as traditional Alpine cattle breeds. Fleckvieh cattle are derived from the Simmental breed and now contain a variable genetic input from Red Holstein and Montbéliard. Cattle of the Swiss Brown breed are derived from the Original Swiss Brown and received genetic input by introgression with semen from the North American Swiss Brown, a breed derived from original Swiss Brown imported from Switzerland in the late 19^th^ and early 20^th^ century.
(TIF)

S3 Fig Proportions of the number of cattle kept per canton and of sequences of BVD viral strains from PI animals per canton. The percentages of cattle hold per canton (black bars) and of the BVD viral sequences (grey bars) analyzed per PI animal detected in a given canton between 2008 and 2011 in relation to total of Switzerland are shown. Cantons (in alphabetical order): AG: Aargau; AI: Appenzell-Innerrhoden; AR: Appenzell-Ausserrhoden; BE: Bern; BL: Basel Country; BS: Basel City; FL: Principality of Liechtenstein (Independent country participating in the Swiss BVD eradication program); FR: Fribourg/Freiburg; GE: Geneva/Genf; GL: Glarus; GR: Graubünden/Grisons; JU: Jura; LU: Luzern/Lucerne; NE: Neuchâtel/Neuenburg; NW: Nidwalden; OW: Obwalden; SG: St. Gallen; SH: Schaffhausen; SO: Solothurn; SZ: Schwyz; TG: Thurgau; TI: Ticino/Tessin; UR: Uri; VD: Vaud/Waadt; VS: Valais/Wallis; ZG: Zug; ZH: Zürich.
(TIF)

S4 Fig Distribution of BVDV-1 subgenotypes of viral strains from cattle of the Holstein breed. Animals of this breed increased in numbers since the late 1960’s. Shown are selected cantons in a direction from West (Geneva and Neuchâtel) to East (St. Gallen and Glarus). The distribution pattern of subgenotypes of BVDV-1 is similar to that of the viral strains of all PI animals in the same Cantons.
(TIF)

S5 Fig Age distribution of the PI animals per subgenotype of BVDV-1. The age of the PI animals analyzed in the frame of the BVD eradication program in Switzerland in 2008 are indicated per subgenotype of BVDV-1. The age of the animals around sampling time is not normally distributed. The medians are not significantly different between the BVDV-1 subgenotypes (p-value 0.136). The Figure was drawn with the Prism 5.1 software for Windows (Available from: https://www.graphpad.com/).
(TIF)
