# Supplementary material for: Traces of history conserved over 600 years in the geographic distribution of genetic variants of an RNA virus: Bovine viral diarrhea virus in Switzerland
Source: PLoS One. 2018 Dec 5;13(12):e0207604. doi: 10.1371/journal.pone.0207604 (PMC6281212; doi:10.1371/journal.pone.0207604)
Supplement: S1 Appendix — (DOCX) [file pone.0207604.s001.docx]

**S1 Appendix. Sequences used for assignment of the strains to pestivirus species and subgenotypes**

The following sequences were used for the assignment of the strains to a pestivirus species: BDV Italy: AJ829444 and AM900848; BDV Switzerland: JQ994199 and JQ994200; BDV Turkey: AM418427 and AM418428; BDV-1: U65023 and U70263; BDV-2: AB122085 and AF144618; BDV-3: JQ994198 and JQ994201; BDV-4: DQ275622 and DQ361072; BDV-5: EF693985 and EF693995; BDV-6: EF693996 and EF694001; Bungowannah: DQ901402; BVDV-1a: M31182; BVDV-1b: JQ994197; BVDV-1c: AF049221; BVDV-1d: AF298065; BVDV-1e: EU180028; BVD-1f: EU224240; BVDV-1 g: AF298069; BVDV-1 h: JQ994196; BVDV-1i: AF298059; BVDV-1j: U97411; BVDV-1k: AF117700; BVDV-1l: EU180024; BVDV-1m: AF526381; BVDV-1n: AB359930; BVDV-2: EU224242 and U18059; HoBi-like: AH013732 and DQ897641; Chamois: AY738080; CSFV: AF091661 and U90951; Giraffe: JQ994202 and AB040131; Pronghorn Antelope: AY781152; Tunisian sheep virus (TSV): AF461996 and AY453630. For the assignment of the strains to BVDV subgenotypes, the following sequences were used: BVDV-1a: EU180026, M31182, M96751 and U94916; BVDV-1b: AF298070, JQ994197, M96687, and U63479; BVDV-1c: AF049221, AF049222, AY762998 and AY763036; BVDV-1d: AF298065, EU224230, EU224234 and JQ994205; BVDV-1e: AF298058, EU180028, JQ994203, JQ994204, and JQ994207; BVDV-1f: AF298073, EU224221 and EU224240; BVDV-1 g: AF298064, AF298069, EU224236 and U97455; BVDV-1 h: AF298068, JQ994196, and JQ994208; BVDV-1i: AF298059 and FJ493484; BVDV-1j: AB078950, U97411 and U97454; BVDV-1k: AF117700, EU224237 and JQ994206; BVDV-1l: EU180024, KF205306 and KF205307; BVDV-1m: AF526381, GU120245 and GU120257; BVDV-1n:AB042661, AB359930, DQ973181 and GQ495676; BVDV-2: AF298055, AF298063, EU224242 and U18059.
