# Supplementary material for: Traces of history conserved over 600 years in the geographic distribution of genetic variants of an RNA virus: Bovine viral diarrhea virus in Switzerland
Source: PLoS One. 2018 Dec 5;13(12):e0207604. doi: 10.1371/journal.pone.0207604 (PMC6281212; doi:10.1371/journal.pone.0207604)
Supplement: S1 Table — (DOCX) [file pone.0207604.s002.docx]

**S1 Table. GenBank****^(a)^ Accession numbers of strains analyzed**

| Classification | Accession # |
| --- | --- |
| BVDV-1b | MH900623 - MH901290; JQ994197 |
| BVDV-1e | MH901291 - MH903552; JQ994204; EU180028 |
| BVDV-1h | MH903553 - MH907214 |
| BVDV-1k | MH907215 - MH908076 |
| BVDV-1g | JX391993 |
| BVDV-1l | EU180024 |
| BDV | MH908078 - MH908083 |

Footnote: **^(a)^** <https://www.ncbi.nlm.nih.gov/genbank/>
