# Supplementary figures and images for: Traces of history conserved over 600 years in the geographic distribution of genetic variants of an RNA virus: Bovine viral diarrhea virus in Switzerland
Source: PLoS One. 2018 Dec 5;13(12):e0207604. doi: 10.1371/journal.pone.0207604 (PMC6281212; doi:10.1371/journal.pone.0207604)

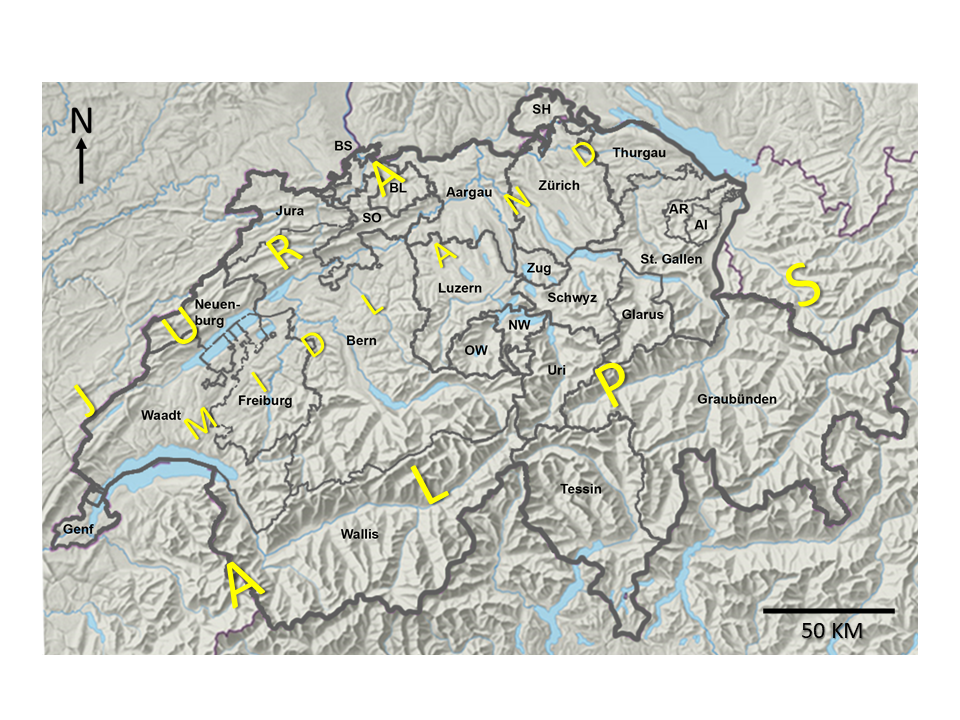

Supplement: S1 Fig — The Swiss Confederation consists of 26 cantons. Note that some of the historic borders between the cantons differ from the current borders indicated in this Figure. Abbreviations: BS: Basel City; BL: Basel Country; SO: Solothurn; OW: Obwalden; NW: Nidwalden; SH: Schaffhausen; AI: Appenzell-Innerrhoden; AR: Appenzell-Ausserrhoden. For a complete list of the cantons, see S3 Fig. (TIF) [file pone.0207604.s004.tif]

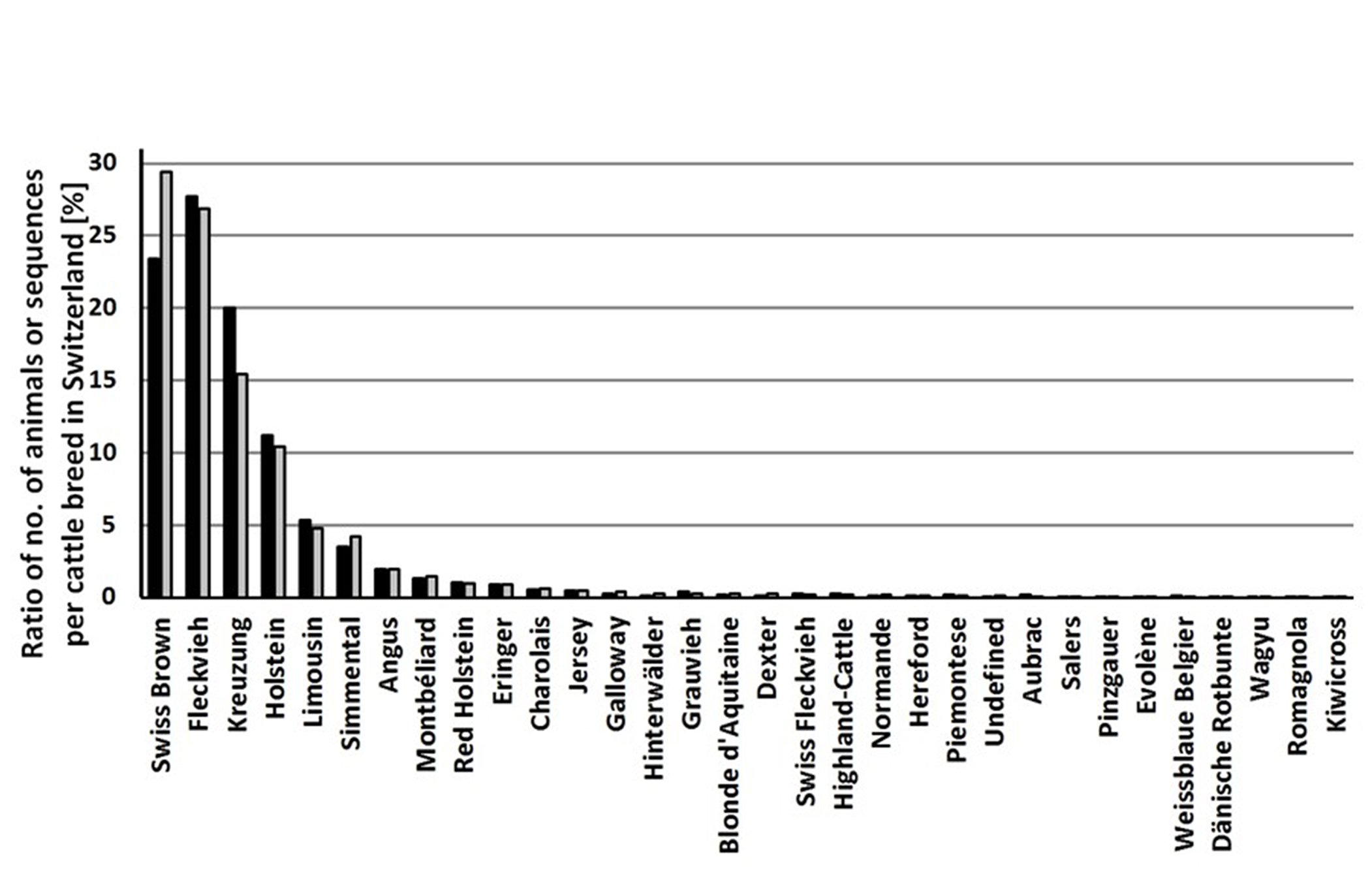

Supplement: S2 Fig — Cattle of different breeds are indicated as a percentage of total cattle population (black bars), and the percentages of BVD viral sequences (grey bars) analyzed per PI animal detected in a given breed between 2008 and 2011 in relation to total of Switzerland are shown. “Kreuzungen” are mostly beef cattle crossbred from different breeds. In addition to Swiss Brown and Fleckvieh, Simmental, Eringer, Hinterwälder, Grauvieh and Evolène are considered as traditional Alpine cattle breeds. Fleckvieh cattle are derived from the Simmental breed and now contain a variable genetic input from Red Holstein and Montbéliard. Cattle of the Swiss Brown breed are derived from the Original Swiss Brown and received genetic input by introgression with semen from the North American Swiss Brown, a breed derived from original Swiss Brown imported from Switzerland in the late 19th and early 20th century. (TIF) [file pone.0207604.s005.tif]

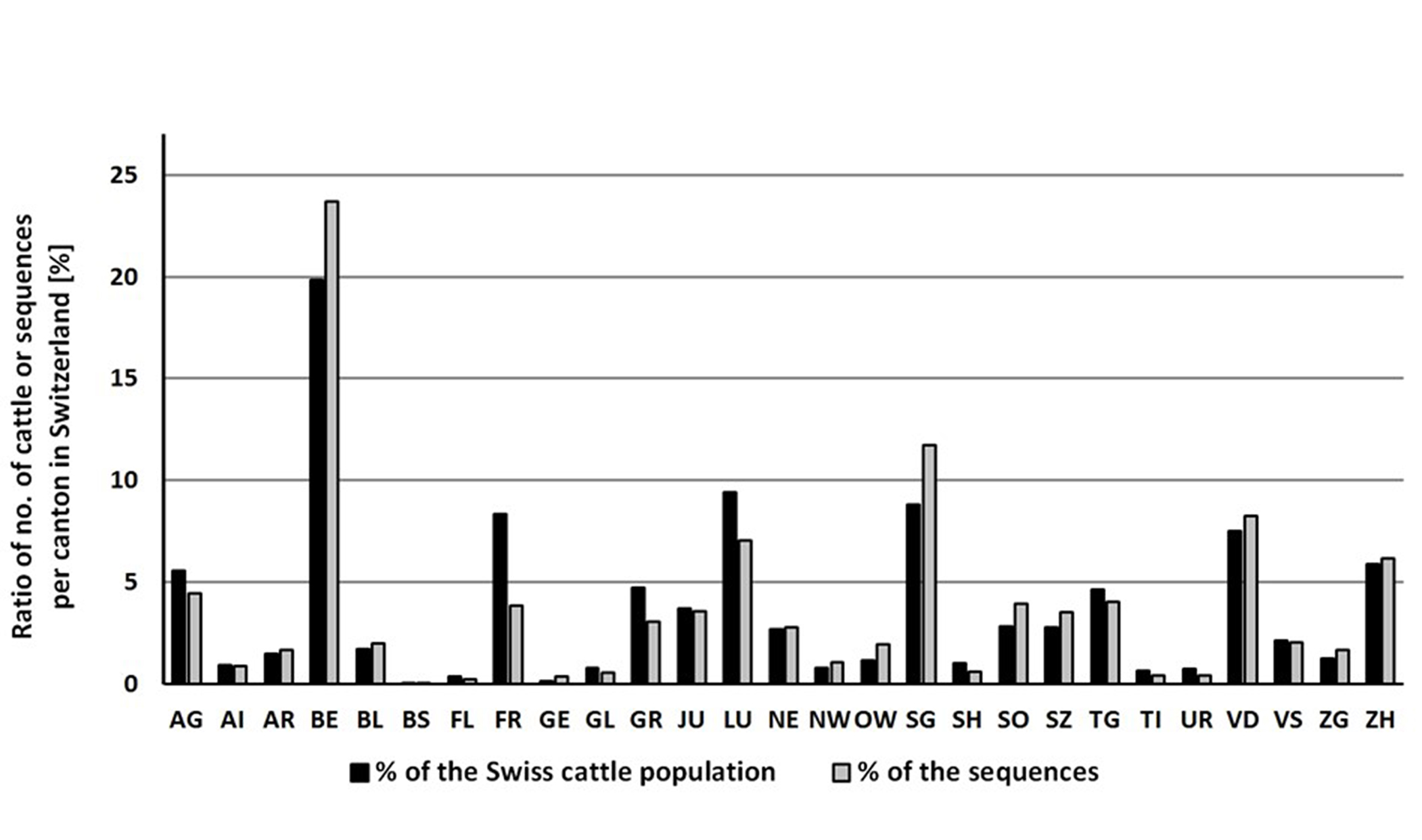

Supplement: S3 Fig — The percentages of cattle hold per canton (black bars) and of the BVD viral sequences (grey bars) analyzed per PI animal detected in a given canton between 2008 and 2011 in relation to total of Switzerland are shown. Cantons (in alphabetical order): AG: Aargau; AI: Appenzell-Innerrhoden; AR: Appenzell-Ausserrhoden; BE: Bern; BL: Basel Country; BS: Basel City; FL: Principality of Liechtenstein (Independent country participating in the Swiss BVD eradication program); FR: Fribourg/Freiburg; GE: Geneva/Genf; GL: Glarus; GR: Graubünden/Grisons; JU: Jura; LU: Luzern/Lucerne; NE: Neuchâtel/Neuenburg; NW: Nidwalden; OW: Obwalden; SG: St. Gallen; SH: Schaffhausen; SO: Solothurn; SZ: Schwyz; TG: Thurgau; TI: Ticino/Tessin; UR: Uri; VD: Vaud/Waadt; VS: Valais/Wallis; ZG: Zug; ZH: Zürich. (TIF) [file pone.0207604.s006.tif]

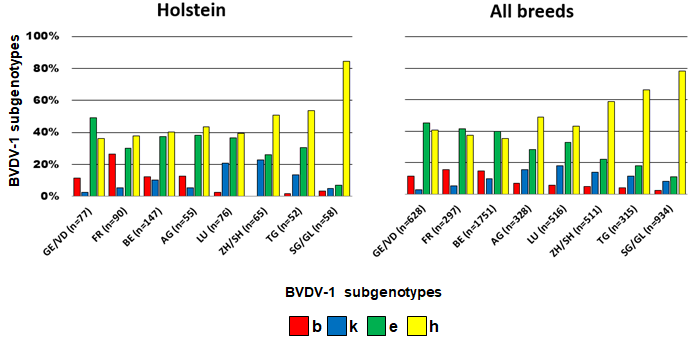

Supplement: S4 Fig — Animals of this breed increased in numbers since the late 1960’s. Shown are selected cantons in a direction from West (Geneva and Neuchâtel) to East (St. Gallen and Glarus). The distribution pattern of subgenotypes of BVDV-1 is similar to that of the viral strains of all PI animals in the same Cantons. (TIF) [file pone.0207604.s007.tif]

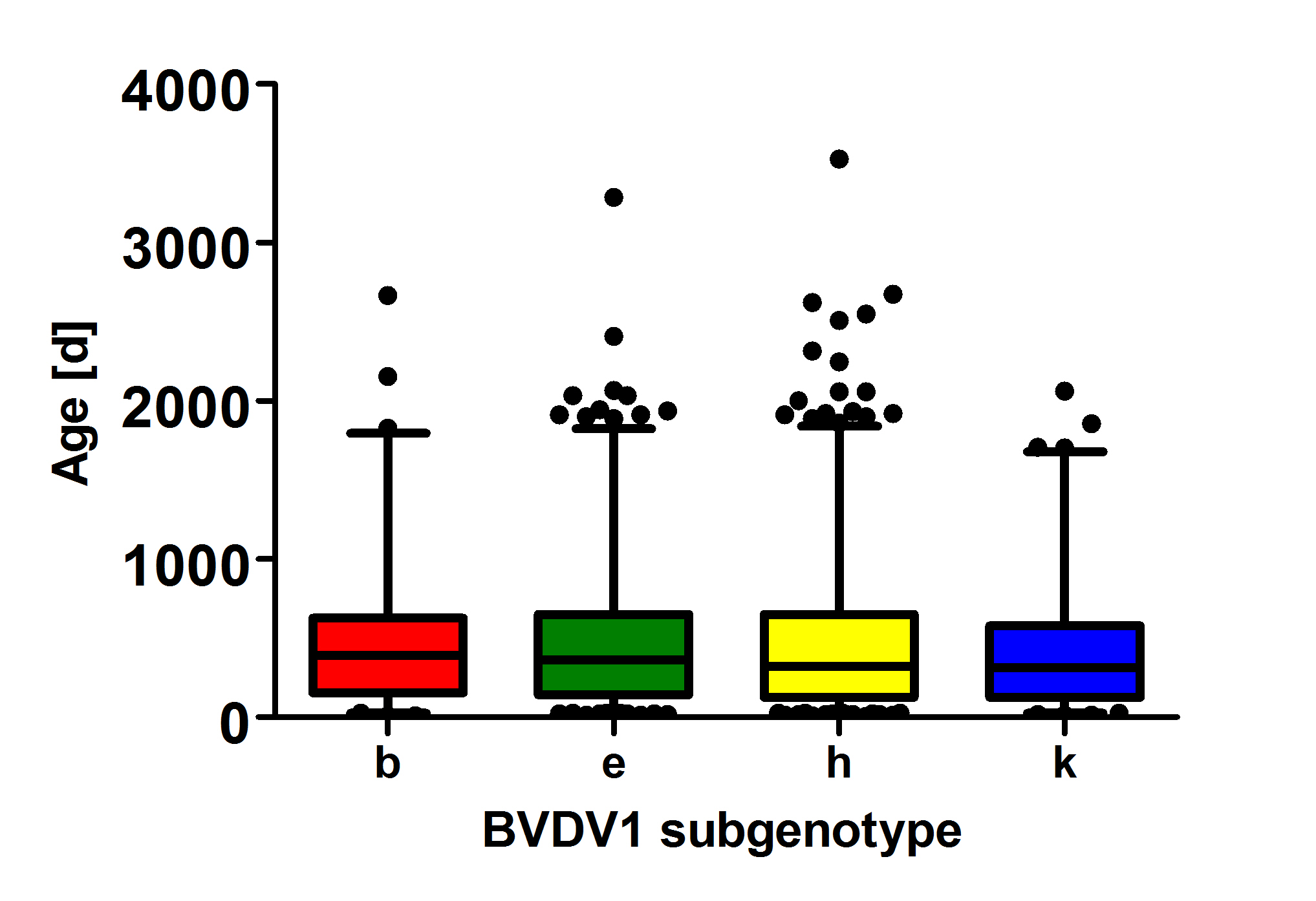

Supplement: S5 Fig — The age of the PI animals analyzed in the frame of the BVD eradication program in Switzerland in 2008 are indicated per subgenotype of BVDV-1. The age of the animals around sampling time is not normally distributed. The medians are not significantly different between the BVDV-1 subgenotypes (p-value 0.136). The Figure was drawn with the Prism 5.1 software for Windows (Available from: https://www.graphpad.com/). (TIF) [file pone.0207604.s008.tif]
