# Supplementary material for: Traces of history conserved over 600 years in the geographic distribution of genetic variants of an RNA virus: Bovine viral diarrhea virus in Switzerland
Source: PLoS One. 2018 Dec 5;13(12):e0207604. doi: 10.1371/journal.pone.0207604 (PMC6281212; doi:10.1371/journal.pone.0207604)
Supplement: S1 Personal Communication — (DOCX) [file pone.0207604.s003.docx]

**S1 Personal Communication. Luzzago and Cerutti**

Dear Prof. Peterhans,

Thank you for your interest in our recent papers.

In Italy, the cattle population is 5.5-6 million heads, mainly Fresian, Piedmontese, Limousine, Charolais, mixed breeds, and much more. Italy  imports 1-1.4 millions cattle/year (see details in attachment)  for reproduction, farming and  fattening, mainly from France, but also from Germany, Ireland, Poland, Belgium, Austria and Slovenia, with no control on BVDV status. On the opposite, the export is reduced.

With such situation, it is then conceivable that in Italy there are more BVDV subtypes than in your country, some high prevalent (BVDV-1b, 1e, 1f) and others restricted to less individuals and/or areas.

We hope that our information may help you in your manuscript.

Kind regards

Camilla Luzzago & Francesco Cerutti

Camilla Luzzago  DVM PhD

Dipartimento di Medicina Veterinaria

Università degli Studi di Milano
Via Celoria 10
20133 Milano
Italia
